# Prognostic Model of Typical Complications Caused by Transcatheter Aortic Valve Replacement

**DOI:** 10.17691/stm2020.12.2.03

**Published:** 2020

**Authors:** E.A. Ovcharenko, K.U. Klyshnikov, V.I. Ganyukov, A.A. Shilov, I.E. Vereshchagin, I.N. Sizova, R.S. Tarasov, L.S. Barbarash

**Affiliations:** Head of Laboratory, Department of Experimental and Clinical Cardiology, Research Institute for Complex Issues of Cardiovascular Diseases, 6 Sosnovy Blvd, Kemerovo, 650002, Russia;; Researcher, Department of Experimental and Clinical Cardiology, Research Institute for Complex Issues of Cardiovascular Diseases, 6 Sosnovy Blvd, Kemerovo, 650002, Russia;; Head of Laboratory, Department of Multifocal Atherosclerosis, Research Institute for Complex Issues of Cardiovascular Diseases, 6 Sosnovy Blvd, Kemerovo, 650002, Russia;; Senior Researcher, Department of Multifocal Atherosclerosis, Research Institute for Complex Issues of Cardiovascular Diseases, 6 Sosnovy Blvd, Kemerovo, 650002, Russia;; Junior Researcher, Department of Multifocal Atherosclerosis, Research Institute for Complex Issues of Cardiovascular Diseases, 6 Sosnovy Blvd, Kemerovo, 650002, Russia;; Senior Researcher, Department of Cardiovascular Disease Diagnostics, Research Institute for Complex Issues of Cardiovascular Diseases, 6 Sosnovy Blvd, Kemerovo, 650002, Russia;; Head of Laboratory, Department of Multifocal Atherosclerosis, Research Institute for Complex Issues of Cardiovascular Diseases, 6 Sosnovy Blvd, Kemerovo, 650002, Russia;; Professor, Member of the Russian Academy of Sciences, Chief Researcher, Research Institute for Complex Issues of Cardiovascular Diseases, 6 Sosnovy Blvd, Kemerovo, 650002, Russia

**Keywords:** transcatheter aortic valve replacement, cardiac conduction disturbances, paraprosthetic regurgitation, discriminant analysis

## Abstract

**The aim of the study** was to develop a prognostic model based on statistical discriminant analysis to assess the risk of postoperative disturbance of cardiac conduction and paraprosthetic regurgitation after transcatheter aortic valve replacement.

**Materials and Methods:**

Clinical data of 10 patients implanted with CoreValve^TM^ prostheses (Medtronic Inc., USA) were used to develop prognostic models. To that end, we analyzed changes in hemodynamic and functional parameters provided by echocardiography in the pre- and postoperative periods.

**Results:**

We observed significant positive changes in the severity of left ventricular myocardial hypertrophy; on the contrary, volume indicators did not significantly change, which might be associated with the concentric type of left ventricular hypertrophy. The discriminant analysis made it possible to determine major (preoperative) morphological and functional indicators associated with the two most common complications of the procedure: left bundle branch block and paraprosthetic regurgitation. Left ventricular posterior wall thickness, interventricular septal thickness, left atrium dimension, and myocardial mass are the critical factors that determine the development of these complications.

**Conclusion:**

In the prognostic model, the proposed weighting coefficients allow one to assess the risk of postoperative complications; however, the presence of false-positive results requires further refinement of these coefficients within the linear equation.

## Introduction

Transcatheter aortic valve replacement (TAVR) is becoming the gold standard for treating severe aortic stenosis in patients with contraindications for surgical aortic valve replacement (SAVR) [1]. In the recent decade, a series of randomized clinical trials comparing TAVR and SAVR were performed. In those, mortality and incidence of stroke were found comparable between these surgical treatments; in addition, significantly fewer complications were associated with TAVR [2, 3]. This progress can be explained by the increasing experience of interventional cardiologists, the optimized design of transcatheter valve prostheses and their delivery systems, and the wider use of three-dimensional (3D) imaging, including multispiral computed tomography (MSCT) at the stage of TAVR planning. All these factors contributed to the expansion of indications for using of TAVR technology in patients with medium and low surgical risk [4] and in patients with a bicuspid valve [5, 6]. Nevertheless, despite the rapid development of this technology, complications typical of this procedure remain: first of all, disturbances of cardiac conduction and paraprosthetic regurgitation [7]. An integrated approach to preoperative planning of TAVR that incorporates morphological and functional analysis can become a valuable tool for reducing the incidence of complications. We, therefore, analyzed the anatomical and hemodynamic changes in patients before and after the TAVR procedure.

**The aim of the study** was to develop a prognostic model based on statistical discriminant analysis to assess the risk of postoperative disturbance of cardiac conduction and paraprosthetic regurgitation after transcatheter aortic valve replacement.

## Materials and Methods

***Clinical data.*** A single-center retrospective study was conducted from August 2014 to November 2018 at the Research Institute for Complex Issues of Cardiovascular Diseases (Kemerovo). We analyzed a number of clinical parameters in 10 patients undergoing TAVR procedures (CoreValve^TM^ bioprosthesis; Medtronic Inc., USA) for aortic stenosis without hemodynamically significant regurgitation. Transfemoral access was used for the catheterization. Additional data were obtained by transthoracic echocardiography (TTE) before and after TAVR using a Vivid 7 Dimension ultrasound diagnostic scanner (General Electric, USA) and a sector phased M4S-RS sensor (1.5–3.6 MHz). The following indicators were studied: end-diastolic diameter (EDD), end-systolic diameter (ESD), ejection fraction (EF), end-diastolic volume (EDV), end-systolic volume (ESV), left atrium (LA) dimension, right ventricular (RV) dimension, interventricular septal (IVS) thickness, left ventricular posterior wall (LVPW) thickness, ascending aorta diameter, stroke volume (SV), myocardial mass (MM), and the maximal trans-prosthesis gradient (Pmax). We analyzed coded patient records that specified the diagnosis, clinical and demographic parameters, surgical risks according to EuroSCORE II, etc. The study was conducted in accordance with the Helsinki Declaration (2013) and approved by the Ethics Committee of the Research Institute for Complex Issues of Cardiovascular Diseases. Informed consent was obtained from each patient.

***The prognostic model.*** To build a prognostic model based on TTE-revealed hemodynamic and functional changes caused by TAVR, a discriminant analysis with stepwise inclusion of quantitative variables was performed. Given the prognostic nature of discriminant analysis, preoperative TTE results were taken as input data. Two “soft” endpoints served as a grouping variable — the presence of a left bundle branch block (LBBB) and the presence of paraprosthetic regurgitation after the valve implantation. The quality of the analysis was evaluated by the significance level p<0.05, by the square of the Mahalanobis distance between two groups, and by re-classification of the available data sample using the obtained linear equation of the discriminant function — verification with determination of errors. The discriminative power of each of the parameters was evaluated by the Wilkes λ index, which, in essence, is an analogue of the partial correlation.

In addition, we determined the weighting coefficients of the linear canonical discriminant function:

$$y=\sum_{i=1}^{N}a_{1}\cdot x_{1}+a_{2}\cdot x_{2}+\ldots +a_{i}\cdot x_{i}+C,$$

where *a_i_* is the weighting coefficient of the *i-*th parameter, *x_i_* is the value of the *i-*th parameter, and *C* is a constant.

***Statistical analysis.*** The obtained data were analyzed using the Statistica v. 10.0 software (StatSoft Inc., USA). The sample distribution mode was evaluated using the Shapiro–Wilk test. It was found that for most indicators the distribution deviated from normal (p<0.01); therefore all descriptive statistics are presented as the median, 25^th^ and 75^th^ percentiles, maximum and minimum. For the pairwise comparison of the results, considering that the samples were dependent variables, we used the Wilcoxon W-test; the differences were considered significant at p<0.05.

## Results

The general characteristics of patients before the TAVR procedure are presented in Table 1. All patients included in the study demonstrated a successful outcome without intra-hospital mortality or the need for repeated aortic valve replacement (Table 2).

**Table 1:** Clinical and demographic characteristics of the patients

Characteristic	Value
Quantity (abs. number)	10
Sex (abs. number/%):	
male	5/50
female	5/50
Age (years)*	79.5 [67; 72.25; 80.75; 84]
The maximum trans-prosthesis gradient (mm Hg)*	77.5 [65.0; 74.0; 101.0; 115.0]
Prior transdermal coronary interventions (abs. number/%)	3/21.4
Left ventricular ejection fraction (ml)*	63 [52; 56; 69; 71]
Angina pectoris I–IV FC (abs. number/%)	4/40.0
Ischemic heart disease (abs. number/%)	8/80.5
Pulmonary hypertension (abs. number/%)	7/70.0
Surgical risk by the EuroSCORE II*	2.9 [2.3; 2.8; 3.6; 7.5]
Concomitant diseases (abs. number/%):	
arterial hypertension	9/90.0
chronic lower limb ischemia	1/10.0
significant stenosis of the internal carotid artery	2/20.0
history of acute cerebrovascular accident	1/10.0
diabetes	4/40.0
chronic kidney disease above stage III	3/30.0
chronic obstructive pulmonary disease	3/30.0
chronic renal failure	2/20.5
Sizes of CoreValve^TM^ (abs. number/%):	
26 mm	5/50.0
29 mm	5/50.0
Post-surgery follow up (years), M±m	0.57±0.25

* Data are presented as Me (min; 25; 75; max).

**Table 2:** Intra- and postoperative interventions

Characteristic	Value
Artificial lung ventilation (h)*	4.5 [3.0; 3.5; 7.0; 7.5]
Inotropic support	0
Stay in the intensive care unit (days)*	1.0 [1.0; 1.0; 2.0; 2.5]
Duration of hospital stay (days)*	10.5 [8.0; 8.0; 12.0; 13.0]
Successful procedures (abs. number/%)^#^	10/100
Intra-hospital mortality	0
Repeated procedure	0
Paraprosthetic regurgitation, grade ≥II (abs. number/%)	3/30
Left bundle branch block (abs. number/%)	4/40
Myocardial infarction	0
Hydrothorax (abs. number/%)	1/10
Atrial fibrillation	0
Pacemaker implantation	0
Vascular access complications	0

* Data are presented as Me [min; 25; 75; max]; ^#^ according to the criteria of the Valve Academic Research Consortium-2 [8].

***Statistical processing.*** The analysis showed significant differences (p<0.05) between the measurements taken before and after the intervention; these changes reflected the process of heart remodeling that was primarily expressed by changes in the left ventricular (LV) volume: relative wall thickness (RWT), IVS thickness, LVPW thickness, MM, SV, as well as Pmax and the ascending aorta diameter (Table 3). The statistical significance of these changes indicated positive dynamics in myocardial remodeling: a decrease in myocardial mass and wall thickness, i.e. a reduction in LV hypertrophy.

**Table 3:** Heart remodeling indicators obtained by transthoracic echocardiography

Parameters	Before TAVR					After TAVR					p
	Me	Min	Max	25%	75%	Me	Min	Max	25%	75%	
End-diastolic diameter	5.7	4.7	6.9	5.2	6.1	5.7	4.7	6.7	5.1	6.3	0.86
End-systolic diameter	3.7	3.0	5.0	3.3	3.8	3.7	3.0	5.6	3.3	4.1	0.26
Ejection fraction	63.0	52.0	71.0	54.0	70.0	63.0	33.0	73.0	45.0	66.0	0.44
End-diastolic volume	157.0	102.0	247.0	130.0	187.0	157.0	102.0	231.0	124.0	201.0	0.74
End-systolic volume	56.5	35.0	118.0	41.0	62.0	56.5	35.0	154.0	44.0	74.0	0.22
Left ventricular relative wall thickness	**0.44**	**0.38**	**0.40**	**0.49**	**0.60**	**0.43**	**0.33**	**0.38**	**0.48**	**0.55**	**0.05**
Left atrium dimension	4.7	4.1	5.8	4.4	4.9	4.6	3.7	6.3	4.5	5.0	0.95
Right ventricular dimension	1.9	1.6	2.8	1.8	2.1	2.0	1.7	3.1	1.8	2.2	0.22
Interventricular septal thickness	**1.4**	**1.1**	**1.6**	**1.3**	**1.5**	**1.3**	**1.0**	**1.5**	**1.2**	**1.4**	**0.05**
Left ventricular posterior wall thickness	**1.4**	**1.1**	**1.6**	**1.3**	**1.5**	**1.3**	**1.0**	**1.4**	**1.2**	**1.3**	**0.05**
Ascending aorta diameter	**3.6**	**3.0**	**4.3**	**3.3**	**4.1**	**3.4**	**2.6**	**4.0**	**3.0**	**3.5**	**0.02**
Stroke volume	107.5	64.0	129.0	79.0	122.0	92.0	52.0	127.0	77.0	105.0	0.33
Myocardial mass	**342.0**	**261.0**	**497.0**	**322.0**	**421.0**	**337.5**	**200.0**	**409.0**	**237.0**	**367.0**	**0.05**
Maximum trans-prosthesis gradient	**77.5**	**65.0**	**115.0**	**70.0**	**93.0**	**11.5**	**7.0**	**17.0**	**11.0**	**16.0**	**0.01**

Notes: statistically significant differences between the measurements made before and after TAVR are shown in bold. The indicator of the relative thickness of the LV walls is estimated as (LVPW thickness + IVS thickness) / EDD.

***Discriminant analysis.*** According to our analysis, the risk of LBBB and the risk of paraprosthetic regurgitation after TAVR as predicted from the TTE parameters were similar. In the analysis, we identified the main parameters having the greatest power of prediction regarding these two complications (Table 4). In this table, the parameters are arranged in decreasing order of their impact on the prognostic function; the most contributing parameters were three anatomical characteristics of the left heart — IVS, LVPW, and LA, and the only functional characteristic — SV. The size of the transcatheter prosthesis, MM, and the surgical risk by EuroSCORE II had the lowest (although statistically significant) contribution to the prognostic function. The rest of the TTE parameters and the clinical and demographic characteristics of patients had no significant power of prediction.

**Table 4:** Key discriminative parameters of the post-TAVR complications

Left bundle branch block		Paraprosthetic regurgitation	
Parameters	Wilkes λ	Parameters	Wilkes λ
Left ventricular posterior wall thickness	0.322	Stroke volume	0.076
Stroke volume	0.210	Right ventricular dimension	0.043
Interventricular septal thickness	0.288	Left ventricular posterior wall thickness	0.013
Left atrium dimension	0.144	Ascending aorta diameter	0.089
Prosthesis size	0.107	Prosthesis size	0.019
EuroSCORE II surgical risk	0.011	Myocardial mass	0.004
Mahalanobis distance	744.72	Mahalanobis distance	4890.33
p-value related to the entire function	0.01	p-value related to the entire function	0.01
Classification errors (%)	10.0	Classification errors (%)	0

Based on this selection, the weighting coefficients of the linear discriminant function were obtained (Table 5). These coefficients are the elements a_1_, a_2_, a_3_, a_4_, a_5_, and a_6_ of the canonical discriminant function. To calculate the risk of LBBB or paraprosthetic regurgitation, one has to multiply the weighting coefficients a_1_–a_6_ in pairs by the parameter values obtained from the preoperative TTE (based on Table 5), and then put the result into the indicated equation. If the final value is less than zero, one can predict the occurrence of complications and, conversely, with a positive final value — no block or regurgitation is expected.

**Table 5:** Linear discriminative function coefficients

Left bundle branch block			Paraprosthetic regurgitation		
Parameters	Weighting coefficient value	Weighting coefficient number	Parameters	Weighting coefficient value	Weighting coefficient number
Left ventricular posterior wall thickness	23,672.6	a_1_	Stroke volume	6.711	a_1_
Stroke volume	–15.2	a_2_	Right ventricular dimension	122.174	a_2_
Interventricular septal thickness	–19,506.7	a_3_	Left ventricular posterior wall thickness	–917.906	a_3_
Left atrium dimension	945.2	a_4_	Ascending aorta diameter	306.685	a_4_
Prosthesis size	–252.7	a_5_	Prosthesis size	–49.983	a_5_
EuroSCORE II surgical risk	–28.6	a_6_	Myocardial mass	0.258	a_6_
Constant	–976.2	C	Constant	372.771	C

Note: the number of the weighting coefficient is given for the canonical form of the discriminant function.

## Discussion

***Statistical analysis.*** The pair-wise statistical comparison showed significant differences in LV myocardial parameters between the measurements made before and after the TAVR procedure: those related to IVS, LVPW, and MM. Along with that, almost all LV volume parameters (EDV, ESV, EF, etc.) did not significantly change after the intervention. The reason for these differences may lie in the nature of the aortic defect, i.e., stenosis without hemodynamically significant regurgitation. This pathology leads to the development of compensatory concentric hypertrophy, associated primarily with thickening of the myocardial wall without significant changes in the heart cavities volume. The RWT indicator precisely confirms the concentric model — in this study, the median RWT value was 0.44 at a threshold value of 0.42, which indicated a significant increase in the thickness of LV walls relative to the cavity volume. After the TAVR procedure, the cause of hemodynamic disturbances — valve stenosis with a high peak gradient — is eliminated; the resulting heart remodeling reduces the myocardial mass, but barely changes the dimensions of the heart cavities, which initially were close to normal (60–130 mm). It is noteworthy that such an effect can occur only if two conditions are met: 1) the initial stenosis of the aortic valve does not cause regurgitation and 2) the valve defect is functionally compensated. Condition 1 is associated with concentric hypertrophy, i.e., a change in the mass, but not the volume of the left ventricle; condition 2 is determined by the compensatory mechanism, where a significant increase in LV volume (development of eccentric hypertrophy) is possible only after a compensation “failure”. In the present study, both conditions were present in all patients, which contributed to the statistical significance of the results (see Table 3).

Our results are in good agreement with similar works by other authors. Thus, a wide range of TTE parameters measured before and after TAVR (Stangl et al. [9]), showed significant changes in IVS, LVPW, and MM — and no changes in volume indicators — EF (only for men) and EDD. In a study of Rymuza et al. [10] in patients with the RWT of 0.6±0.1 cm, EDD, EDV, and EF did not differ between the pre- and postoperative periods (12 months) due to the same reasons — the initial presence of both concentric and compensated hypertrophy.

***Discriminant analysis.*** Our discriminant analysis revealed a significant contribution of anatomical indicators to the risk of both complications — LBBB and regurgitation, which, apparently, requires a more thorough analysis of these phenomena. In the current practice of preoperative planning, it is recommended (2017 ESC/EACTS guidelines) to use MSCT to assess the anatomy and size of the aortic root, the shape and size of the valve annulus, its distance to the mouth of the coronary arteries, the distribution of calcifications and the number of aortic valve cusps [1]. In addition, according to this recommendation, MSCT is more preferable than TTE because the latter highly depends on the operator and may also have sub-optimal image quality. However, the present study shows that the results of TTE, which primarily reflect the anatomy of the left heart (LVPW, IVS, LA), also have important prognostic values.

It is noteworthy that, in addition to the anatomical characteristics, the size of the prosthesis for TAVR also contributed to the prediction algorithm. The development of LBBB is caused by excessive pressure exerted by the supporting frame of a self-expanding prosthesis on the conduction paths passing near the fibrous ring of the aortic valve [11]. In this study, a significant contribution of the prosthesis size to the risk of both complications was found. The result necessitates a more careful selection of bio-prostheses for implantation, including the new version of Evolut R^ТМ^ (Medtronic Inc., USA) [12].

In paraprosthetic regurgitation, the mechanism is associated with severe valvular calcification, which prevents the prosthesis from full straightening and tight-fitting [13]. Presumably, a smaller prosthesis would produce less force than that needed to shift the calcium deposits to the periphery. Nevertheless, despite its significance the contribution of the size indicator to the final prognostic model is rather small (see Table 4) and should be interpreted with caution.

In general, the obtained linear function coefficients (see Table 5) demonstrate a high power of prediction for LBBB and regurgitation risk. However, we found that in the case of LBBB, the coefficient values had a 10% error, which, according to the statistical analysis (classification matrix) produced false-positive estimates. On the one hand, the presence of such an error can be considered non-critical, since for this type of intervention a false-positive result can be interpreted as additional “insurance” — the model predicts the risk of complications, although in practice it may not happen. On the other hand, the mere presence of a classification error is a negative factor, which does not allow us to definitely recommend this algorithm for decision-making at the preoperative stage. Additionally, it is worth noting that in the present study, the number of patients was limited but uniform in terms of its clinical characteristics (demography, functions, comorbidity). Therefore, the obtained coefficients of the linear function are somewhat “idealized” and may prove less sensitivity/specificity for more heterogeneous groups.

## Conclusion

Statistically significant changes in morphology and function of the left ventricle (primarily, thickness and mass of the myocardium) indicating heart remodeling after transcatheter aortic valve replacement were demonstrated. The obtained discriminative linear function allows one to predict the risk of two major complications of transcatheter aortic valve replacement — left bundle branch block and paraprosthetic regurgitation (p<0.01). This conclusion, although based on the parameters of heart anatomy, should be taken with caution, since (in this study) it produced 10% of false-positive predictions for left bundle branch block.
